# Recent Developments in Delivery of MicroRNAs Utilizing Nanosystems for Metabolic Syndrome Therapy

**DOI:** 10.3390/ijms22157855

**Published:** 2021-07-23

**Authors:** Tong Li, Liye Zhu, Longjiao Zhu, Pengjie Wang, Wentao Xu, Jiaqiang Huang

**Affiliations:** 1 Beijing Advanced Innovation Center for Food Nutrition and Human Health, College of Food Science and Nutritional Engineering, China Agricultural University, Beijing 100083, China; leetong0606@126.com (T.L.); zhuliye89@126.com (L.Z.); zhulongjiao@126.com (L.Z.); Wpj1019@cau.edu.cn (P.W.); xuwentao@cau.edu.cn (W.X.); 2 Key Laboratory of Precision Nutrition and Food Quality, Ministry of Education, Department of Nutrition and Health, China Agricultural University, Beijing 100083, China

**Keywords:** MicroRNAs, metabolic syndrome, nanotechnology, delivery systems

## Abstract

Metabolic syndrome (MetS) is a set of complex, chronic inflammatory conditions that are characterized by central obesity and associated with an increased risk of cardiovascular diseases. In recent years, microRNAs (miRNAs) have become an important type of endocrine factors, which play crucial roles in maintaining energy balance and metabolic homeostasis. However, its unfavorable properties such as easy degradation in blood and off-target effect are still a barrier for clinical application. Nanosystem based delivery possess strong protection, high bioavailability and control release rate, which is beneficial for success of gene therapy. This review first describes the current progress and advances on miRNAs associated with MetS, then provides a summary of the therapeutic potential and targets of miRNAs in metabolic organs. Next, it discusses recent advances in the functionalized development of classic delivery systems (exosomes, liposomes and polymers), including their structures, properties, functions and applications. Furthermore, this work briefly discusses the intelligent strategies used in emerging novel delivery systems (selenium nanoparticles, DNA origami, microneedles and magnetosomes). Finally, challenges and future directions in this field are discussed provide a comprehensive overview of the future development of targeted miRNAs delivery for MetS treatment. With these contributions, it is expected to address and accelerate the development of effective NA delivery systems for the treatment of MetS.

## 1. Introduction

**Figure 1:**
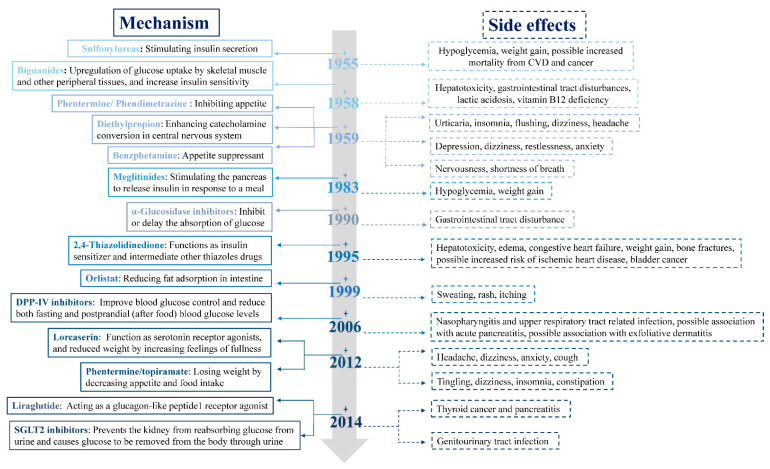
Developments in clinical medication classes associated with MetS management. The mechanism of drugs (**left** panel, solid lines) and their side effects (**right** panel, dashed lines).

Metabolic syndrome (MetS) is a set of complex, chronic inflammatory conditions that are characterized by central obesity and associated with an increased risk of cardiovascular diseases (CVD) and type 2 diabetes mellitus (T2DM) [1]. Owing to poor eating habits and sedentary lifestyles, estimates indicate that greater than 34% of the population will up to standard of MetS, thus, resulting in both public health and clinical problems [2]. Use of some small molecule drugs related to the management of MetS has increased dramatically over the past two decades, yet their side effects are also enormous and still to be far from ideal (Figure 1).

**Figure 2:**
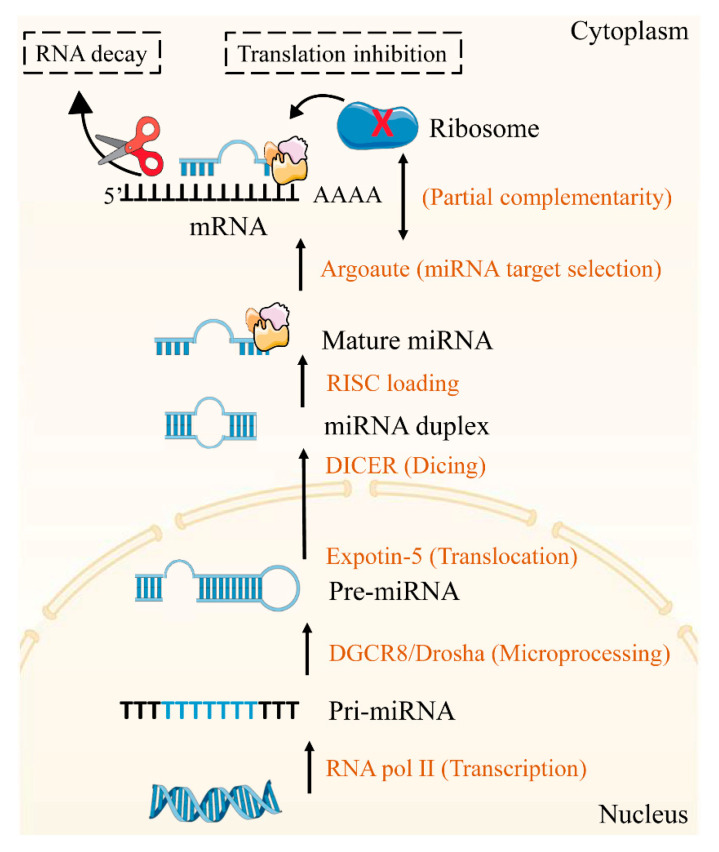
MiRNA biogenesis.

Nucleic acid (NA) therapeutics are the third major category of therapeutics by changing the expression of causative gene(s) to achieve targeted treatment strategies [3]. Among them, microRNAs (miRNAs) as the non-coding endogenous RNAs could bind to the 3′ untranslated regions (UTRs) of mRNA and regulate several genes simultaneously [4] (Figure 2). Up to now, several miRNA-based therapeutics have exerted great effects for some diseases treatment [5,6]. However, the poor targeting ability, short circulation time and immune response of naked miRNA-based agents set a limit to clinical application. Consequently, successful delivery of miRNA requires suitable vectors to protect against nuclease degradation in blood, avoid immune stimulation and target to specific cells. As the nanotechnology develops, nanovectors with sizes between 1 and 200 nm, play key roles in drugs and NA delivery in fighting diseases [7]. Compared with injected naked NA, nanovectors-encapsulated NAs have longer systemic half-lives. Additionally, this encapsulation, along with specific targeting modifications, enables NA to accumulate in local areas, thus, minimizing systemic toxicity [8]. However, fewer studies have focused on the miRNA-based therapies for the management of MetS because the complex pathogenesis and microenvironment of MetS makes it difficult to achieve targeted delivery of miRNA.

This review focus on miRNA-based delivery nanosystems for MetS therapy. First, this review summarizes the miRNAs associated with MetS in different metabolic organs and pathways. Next, it discusses recent advances in the functionalized development of classic delivery systems for NA. Then, this work briefly discusses the intelligent strategies for emerging novel delivery systems for NA delivery. Finally, this review concludes with a summary of the challenges in the field and potential future directions for research and development.

## 2. Current Progress and Advances on miRNAs in the Context of MetS

### 2.1. The Mechanisms of miRNAs

With the discovery of *lin-4* in *Caenorhabditis elegans* from 1993, miRNAs were recognized as an essential molecule for gene regulation at the post transcriptional level [9]. The biogenesis and mechanism of miRNAs are described in Figure 2. Firstly, pri-miRNA are transcribed by RNA polymerase II from genomic DNA. Under the action of Drosha-DGCR8, the pri-miRNA is processed to pre-miRNA and transferred to the cytoplasm by exportin-5 where it is recognized and cleaved by the DICER complex to create a miRNA duplex. Then the mature miRNA assembles into RISC complex after duplex loosened. Depending on the degree of homology between the miRNA sequence to the 3′UTR of the mRNAs, the target mRNAs are cleavage or translation repression.

### 2.2. Therapeutic Target of miRNAs

Presently, it is already known that not only in gene regulation, miRNAs are also closely related to a variety of cell processes and diseases. Dysregulation of miRNAs affected the status and functions of metabolic organs, including the adipose tissue (AT), pancreas, liver, and muscle, possibly contributing to the development of MetS. Study on the relationship between MetS and miRNAs can further clarity the pathogenesis of the MetS, and provide a new direction for its prevention and treatment. The current progress and advances on therapeutic target of miRNAs is summarized in Table 1.

Islets β-cells and insulin play an important role in T2DM, which is a severe metabolic disease characterized by insulin resistance (IR) in peripheral tissues. Declined insulin levels have been attributed to a decrease in β-cell mass and islets dysfunction in regulating glucose homeostasis [10]. Numerous miRNAs have been reported to be involved in pancreatic development and insulin secretion, with some of them directly binding with the key transcription factors and kinases to stimulate insulin secretion, increase insulin sensitivity or regulate islet functions. MiR-375 is the most abundant in pancreatic islets, which maintains the mass and function of pancreatic β-cells. Ouaamari et al. found that miR-375 directly targets 3-phosphoinositide dependent kinase-1 (PDK1), a key molecule in the developments of pancreatic β-cells, and decreases the blood glucose levels [11].

The liver, one of the most important endocrine organs, regulates blood glucose concentration and maintains energy homeostasis. Some studies have demonstrated that miRNAs regulate liver function and are involved in the pathogenic process of MetS, such as nonalcoholic fatty liver disease (NAFLD) and nonalcoholic steatohepatitis (NASH) [12,13]. These effect on MetS associated with the expression of key genes involved in fatty acid metabolism, cholesterol homeostasis and liver functions, including the rate-limiting enzyme 3-hydroxy-3-methylglutaryl-CoA-reductase and ATP-binding cassette A1 (ABCA1). One such example is miR-122, which is a dominant hepatocyte-specific miRNA, is essential for lipid metabolism and has anti-inflammatory activity in the liver [14]. Cheung et al. have identified differentially expressed miRNAs in human NASH, and miR-122 level is significantly diminished in subjects with NASH [15]. Hepatic miR-223 additionally regulates cholesterol biosynthesis in mice and humans by targeting the 3-hydroxy-3-methylglutaryl-CoA synthase 1 and the sterol-C4-methyloxidase-like protein. Moreover, this miRNA inhibits the cholesterol uptake by targeting the scavenger receptor class B member 1 and promotes cholesterol efflux by positively regulating the expression of ABCA1.

In addition, skeletal muscle is the major user of glucose and energy in the human body. When strenuous exercise consumes energy, muscle glycogen is decomposed, thus, producing lactic acid, which is transported to the liver and used for the synthesis of liver glycogen and glucose to provide energy. It has been reported that some miRNAs, which participate in proliferation and metabolism, are detectable in skeletal and cardiac muscle tissues [16]. For example, Feng et al. have determined that miRNA-133a plays a role in mediating altered gene expression and structural/functional deficits in the heart [17]. Additionally, miR-29a is upregulated in the skeletal muscle in intrauterine growth retardation, which induces MetS and is often characterized by IR. Overexpression of miR-29a decreases the levels of glucose transporter 4, thereby partially inducing a decrease insulin-dependent glucose uptake [18].

AT has become an important endocrine system for energy storage, which associated with insulin sensitivity, blood glucose level and inflammation, and participate in pathophysiological processes. In AT, numerous miRNAs contributed to the regulation of energy balance and metabolic homeostasis associated with MetS. Lee et al. have identified miRNAs varying in abundance during the adipocytes differentiation. Among them, miR-130 strongly affects adipocytes differentiation and adipogenesis by repressing the biosynthesis of PPARγ, a major regulator of adipogenesis [19]. MiR-143 is another positive regulator of adipocyte differentiation, acting through ERK5 signaling pathway. Overexpression of miR-143 may impair insulin-stimulating AKT activation and glucose homeostasis in obese mice.

**Table 1:** MiRNAs associated in MetS with metabolic tissues.

Metabolic Tissues	MicroRNAs	Pathway/Process	Targets	References
Pancreas	miR-375	Islet functions	PDX1, HNF6, INSM1, Ngn3	[20,21]
	miR-15a/b, miR-16, miR-195	Islet functions	Ngn3	[22]
	miR-7	Islet cells differentiation	Pax6	[23,24]
	miR-96	Insulin secretion	Stx-1α	[25]
	miR-124a	Insulin secretion	Stx-1α	[25]
	miR-124a	Insulin release	SNAP25, Rab3A, Synapin-1A, Rab27A, Noc2	[25]
	miR-124a	Islet functions	Foxa2, Pdx1, Creb1	[26]
	miR-9	Insulin secretion	Stx-1α	[25]
	miR-9	Insulin release	Onecut-2, Granuphilin/slp4	[27]
	miR-29	Insulin secretion	Stx-1α, Mct1	[28]
	miR-192-5p	Islet β-cell functions	Clock	[29,30,31,32]
	miR-192-5p	Insulin sensitivity	CaV1	[29,30,31,32]
	miR-103, miR-107	Insulin sensitivity	CaV1	[33]
Liver	miR-122	Liver functions	LETFS, HNF6, HNF4a	[34,35]
	miR-122	Lipid metabolism	SREBF1	[34,35]
	miR-26a	Lipid metabolism	ACSL3, ACSL4, PKC, GSK3β, SREBF2	[36]
	miR-33b	Lipid metabolism	SREBF1	[37]
	miR-34a	Fatty acid metabolism	SIRT1	[38]
	miR-34a	Lipid metabolism	SREBP1	[38]
	miR-370	Lipid metabolism	MECPT	[39]
	miR-96, miR-183	Lipid metabolism	SREBP	[40]
	miR-30c	Lipid metabolism	RARB, LPGAT1, MTP	[41,42,43,44]
	miR-30c	Glucose metabolism	IDH1, LIN28B	[41,42,43,44]
	miR-192-5p	De novo lipogenesis	SREBF1, SCD-1	[30,31,32,33]
	miR-192-5p	Inflammation	FoxO1	[30,31,32,33]
	miR-192-5p	Cholesterol homeostasis	ABCG4	[30,31,32,33]
	miR-192-5p	Lipid metabolism	ElOVL1, ElOVL5, PPARA, VLDLR, FABP3, ATF1, CAV2, CRTc2, DBT, IGF1	[30,31,32,33]
	miR-33a-3p	Cholesterol efflux	ABCA1, ABCG1	[45]
	miR-33a-3p	Insulin signaling	IRS2, SIRT6	[45]
	miR-33a-3p	Lipid metabolism	SREBP2, SREBF1	[45]
	miR-223	Cholesterol efflux	ABCA1	[45]
	miR-223	Cholesterol biosynthesis	HMG-CoA, SC4MOL	[46]
	miR-206	Lipid metabolism	LXR2	[46]
	miR-200	Liver cell growth and proliferation	PI3K	[47]
	miR-27a-3p	Cholesterol metabolism	LDLRAP1, LRP6	[48,49,50,51]
	miR-27a-3p	De novo lipogenesis	SCD1, RXRα	[48,49,50,51]
	miR-27a-3p	Inflammation	Nrf2, NF-κB	[48,49,50,51]
	miR-27a-3p	Lipid metabolism	PPARA, FASN, SREBF1, FAS	[48,49,50,51]
	miR-128	Cholesterol metabolism	LDLR	[52]
	miR-128	Cholesterol efflux	ABCA1	[52]
	miR-128	Inflammation	Nrf2	[52]
	miR-130b, miR-301b	Cholesterol metabolism	LDLR	[53,54,55]
	miR-130b, miR-301b	Cholesterol efflux	ABCA1	[53,54,55]
	miR-27b	Cholesterol efflux	ABCA1	[48,49,50,51]
	miR-27b	Lipid metabolism	LDLR	[48,49,50,51]
	miR-140-5p	Cholesterol metabolism	LDLR	[10,55,56]
	miR-140-5p	Inflammation	Nrf2	[10,55,56]
	miR-140-5p	AMPK/SREBP1 pathway	NEAT1	[10,55,56]
	miR-2, miR-148a-3p, miR-185	Cholesterol metabolism	LDLR	[57,58]
	miR-2, miR-148a-3p, miR-185	Lipid metabolism	SERBP2	[57,58]
	miR-21	Lipid metabolism	HMGCR, HOMER1, Smad7	[59,60,61,62,63]
	miR-344	Lipid metabolism (Wnt/β-catenin signaling pathway)	GSK3β	[49]
Muscle	miR-29a	IR	PPARδ,	[64]
	miR-29a	Glucose uptake	IRS-1	[65]
	miR-106b	Mitochondrial dysfunction, IR	mitofusin-2	[66]
	miR-208	Glucose metabolism	MED13	[67,68]
	miR-199a-3p, miR-590-3p	p/Akt pathway	HOMER1, CLIC5	[55,58,69,70,71]
AT	miR-14	Lipid metabolism	p38, MAPK	[72]
	miR-143	Adipocyte differentiation	ERK5 signaling	[73]
	miR-27a, miR-130a	Adipocyte differentiation	PPARγ	[55]
	miR-27b, miR-363	Adipocyte differentiation	C/EBPα, PPARγ	[74]
	Let-7	Cell functions	RAS, HMGA2	[75,76,77]
	Let-7	Glucose metabolism	INSR, IGF1R	[75,76,77]
	Let-7	Adipogenesis	AT-hook2, PPARγ, FABP4	[75,76,77]
	miR-375	Adipocyte differentiation	C/EBPα, PPARγ2	[78]
	miR-206	Lipid accumulation	PPARγ, PTEN, FAS, C/EBPα	[79]
	miR-146b	Metabolic homeostasis	SIRT1	[80]
	miR-8	Adipogenesis	FABP4	[47,81]
	miR-8	Fat body growth and differentiation	PI3K	[47,81]
	miR-210	Adipocyte differentiation (PI3K/Akt pathway)	SHIPI	[82]
	miR-21	Adipocyte differentiation	AP-1, TGF-β receptor 2	[83]
	miR-30c	Adipocyte differentiation	SERPINE1, ACVR1	[41,42,43,44]
	miR-142-5p	Inflammation	Nrf2	[55,56,84]
	miR-26a	Inflammation	IL-6, IL-17	[78,85,86,87]
	miR-26a	Autophagy	BECN1, LC3	[78,85,86,87]
	miR-370	Metabolic homeostasis	CPTIA	[39]
	miR-22	Adipogenesis	HDAC6	[88]
	miR-31	Lipid accumulation	C/EBPα	[89]
	miR-33b	Lipogenesis	EBF1	[37]
	miR-93	Adipogenesis	Sirt7, Tbx3	[90]
	miR-125a	Adipogenesis	ERRα	[91]
	miR-155	Adipocyte differentiation	PPARγ	[92]
	miR-145	Preadipocyte differentiation	IRS1	[93]
	miR-155	Lipid metabolism	C/EBPβ	[94]
	miR-194	Stimulates osteogenesis and inhibits adipogenesis	COUP-TFII	[95]
	miR-224	Fatty acid metabolism	EGR2	[96]
	miR-363	Adipocyte differentiation	E2F3, C/EBPα, PPARγ	[74]
	miR-369	Adipogenic differentiation	FABP4	[97]
	miR-448	Lipid metabolism	KLF5, 5-HT2AR, 5-HT2CR	[98]
	miR-709	Lipid metabolism (Wnt/ß-catenin signaling)	GSK3β	[99]
	miR-637	Adipogenesis	Sp7	[100]
	miR-320	Adipogenesis	RUNX2	[101]
	miR-199a	Adipogenesis	Smad1	[70]
	miR-103	Adipogenesis (AKT/mTOR signal pathway)	MEF2D	[102]
	miR-26b	Adipogenic differentiation	PTEN	[79,87]

### 2.3. Therapeutic Potential of miRNAs

Due to the miRNAs’ ability to simultaneously affect multiple pathways/gene networks, including insulin signaling pathway, glucose and lipid metabolism, miRNA-based treatments for MetS hold great promise in clinical trials. According to the unusual expression of miRNAs in some metabolic organs, antisense inhibitors of overexpressed mature miRNA sequence can be used as a repair technique to suppress overexpression of targeted genes. Additionally, using synthetic mimics of the targeted mature miRNA could restore the down-expression of miRNA in MetS. Normal cellular function can be restored by resetting miRNA expression and exogenously providing mature miRNA.

### 2.4. Challenges in miRNA-Based Treatment

With the progress of gene therapy, miRNAs have received wide-spread attention. MiRNA-based treatment may help in MetS therapy but will not be a panacea all the time. Apart from suppressing genes expression, miRNAs can also induce genes activation in some cases and even cause pseudo activity. The instable structure and low molecular mass of miRNAs could rapidly degraded by a variety of nucleases in body fluids and quickly eliminated by kidney filtration. In addition, delivering naked miRNAs to targeted cells were hindered uptake due to negatively charged groups of miRNAs, that will greatly reduce the therapeutic effects. The successful targeted delivery and gene regulation by miRNAs is a complex and challenging process, requiring suitable vectors with high encapsulation efficiency (EE) of miRNAs and ensure efficient and timely release. Due to the small sizes and ease of surface modification, the nanocarriers could link with miRNAs leading to a protection from miRNAs degradation and long circulation. Development of new perspectives for nanocarriers design accelerate the transition from basic research to clinical applications of miRNA-based treatment.

## 3. Exosomes: The Natural Nanocarrier

**Figure 3:**
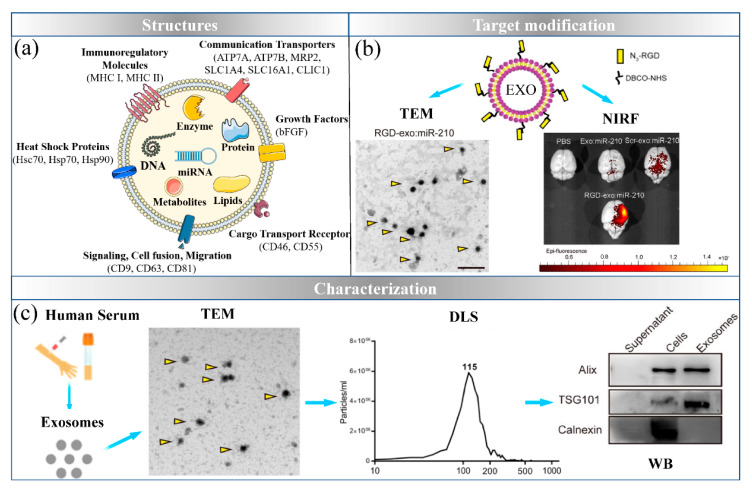
Exosomes-based delivery system. (**a**) The structure of exosomes; (**b**) the targeted modifications of exosomes (reproduced with permission from copyright 2019 BioMed Central); (**c**) exosomes are characterized by three levels (reproduced with permission from copyright 2019 BioMed Central).

Exosomes are vesicles 40–100 nm in size, which carry genetic and proteomic information and are released from cells to mediate cellular communication for pathophysiological processes [83]. Exosomes are derived from the endosomal system, the released exosomes are transported to the recipient cells through ligand–receptor binding, budding-fusion or internalization. As shown in Figure 3a, the lipid bilayer surfaces of exosomes contain proteins including the family of heat shock proteins, the family of tetraspanins, membrane adhesion proteins, transporter proteins and receptors, thus, conferring functions in cell fusion, adhesion and migration. Therefore, exosomes could serve as a natural delivery system.

### 3.1. Sources and Targets for Engineered Exosomes

#### 3.1.1. Peripheral Blood

Normalized blood circulation maintains physiological homeostasis and carries valuable information about the human body. In theory, the detection of DNA, RNA and vesicles carried by the blood can aid in the diagnosis and monitor various diseases. Peripheral blood is a common source for engineered exosomes owing to the obtainability. However, isolation of exosomes and other extracellular in the blood is time consuming. Additionally, the specific source cells and the targeting of exosomes in blood remain unclear.

#### 3.1.2. Mesenchymal Stem Cells (MSCs)

MSCs is one of the pluripotent stem cells that can self-renew and differentiate into multiple tissues. They are present in multiple tissues, including umbilical cord, bone marrow and AT. Owing to exosomes are an important part of paracrine MSCs, the MSCs-derived exosomes can participate in cells communication by transferring information to targeted cells and maintaining the same biological activity as mesenchymal stem cells. Previous studies have found that the MSCs-derived exosomes show high differentiation potential and high survival rates after transplantation, and thus, may play an important role in anti-cardiomyocyte apoptosis and anti-inflammation [85]. However, isolation and purification of MSCs-derived exosomes are difficult, thus limiting their use in large scale samples.

#### 3.1.3. Adipocytes

AT is an important energy metabolism organ in the human body. Increasingly, studies are focusing on adipocyte-derived exosomes, which act on tissues and organs throughout the body in an autocrine, paracrine and endocrine manner, thereby maintaining the crosstalk between organs, including the liver and skeletal muscle cells [86]. In addition, adipocyte-derived exosomes lead to IR and T2DM, which act on immune cells in AT [87]. Investigating adipocyte-derived exosomes facilitate further exploration of the pathogenesis of MetS.

#### 3.1.4. Immune Cells

Immature dendritic cells provide the best option for generating exosomes from human or animal resources, because these exosomes have the lowest levels of surface biomarkers, such as a MHC-I, MHC-II, CD40 and CD86, thus, diminishing the immune response [88]. Therefore, application of the exosomes derived from immune cells is safe in clinical activity.

#### 3.1.5. Milk

In addition to being present in blood, urine and other body fluids, miRNAs are also present in milk, which is enriched in exosomes. Some studies have confirmed that milk-derived exosomes resist gastrointestinal digestion and are absorbed by intestinal epithelial cell [89]. The functional analysis of milk-derived exosomes is expected to be the next research hotspot in food nutrition and biomedicine, and the function of milk-derived exosomes form different animals must be confirmed by additional data.

### 3.2. Exosomes Delivery of miRNAs for MetS Treatment

Various strategies are available for loading miRNAs into engineered exosomes, such as incubation, sonicating, extrusion, electroporation, antibody binding, saponin-assisted, click chemistry and freezing. Among them, electroporation is a common method for loading large molecules such as miRNAs. Alternatively, sonication is suitable for loading miRNAs into exosomes, owing to high loading efficacy and rapidity. As a natural delivery system, exosomes loaded miRNAs and delivered into the bloodstream to influence metabolism. Due to the abundance and obtainability of blood, exosomes derived from peripheral blood can be isolated in large quantities for miRNA delivery. Kang et al. have developed that miR-21 inhibitor loaded into human peripheral blood-derived exosomes, which have been found to decrease fibrotic remodeling in a myocardial infarction mouse model through suppression of SMAd family member 7 [91]. In addition, Ying et al. have developed that miR-155 inhibitors packaged into AT macrophages exosomes for insulin sensitivity and insulin signaling enhancement in obese mice related to the target gene PPARγ [92].

Ischemic stroke is recognized as one of the serious cardiovascular diseases, due to the blood vessels in brain are blocked and ruptured by plaque. Some nanocarriers have been found to have difficultly crossing the blood−brain barrier (BBB) and targeting ischemic neurons. Zhang et al. have developed that cholesterol-modified miR-210 loaded into mesenchymal stromal cell-derived exosomes with c(RGDyK) peptide modifications [90]. This functionalized strategy induced the lesion region accumulation of miR-210 and consequently to promote microvascular angiogenesis. Similarly, the blood placental barrier formed by the mother’s endometrium and the fetal chorion is also a major challenge for miRNA-based nanocarriers delivery. Studies have shown that obesity of pregnant mice obesity during pregnancy in pregnant mice may affect the development of offsprings and even lead to abnormal pregnancy outcome. As natural cell vesicles, exosomes have the ability to cross biological barriers, but more data are needed to prove the safety of miRNA delivered by exosomes on offsprings.

### 3.3. Challenges in Exosomes-Based Delivery System

Compared to conventional nanocarriers, exosomes can possibly bypass the endosomal pathway and lysosomal degradation and deliver miRNAs directly into cytoplasm. The non-immunogenicity of exosomes makes the possibility of repeated administration in MetS treatment. To date, there is no clear ideal purification technique to isolate exosomes with high purity and a wide range of separation technologies are costly for a large-scale production. Besides, functionalized surface modifications of engineered exosomes by chemical bonds need to be examined for safety and efficacy. Clearly, further research is required into the composition of exosomes and their efficacy as a delivery system.

## 4. Liposomes: Simulating Biological Membrane

With the discovery of lipid-based nanoparticles from 1964, liposomes as one of the successful delivery systems applied in commercial transfection reagents and drugs delivery, because of the lipids components are easily taken up by cells and their low toxicity. Liposomes are generally 50–300 nm vesicles formed by self-assembly of one or more bilayers of natural or synthetic lipids in solution, such as phospholipids. Typically, the orderly structure which miRNAs loaded in the inner aqueous core or between the lipid bilayers makes liposomes the unique ability to protect from enzymatic degradation. To date, many lipid-based nanoformulations have been used for the treatment of various diseases.

### 4.1. Design Strategies of Lipid-Based Delivery Systems

**Figure 4:**
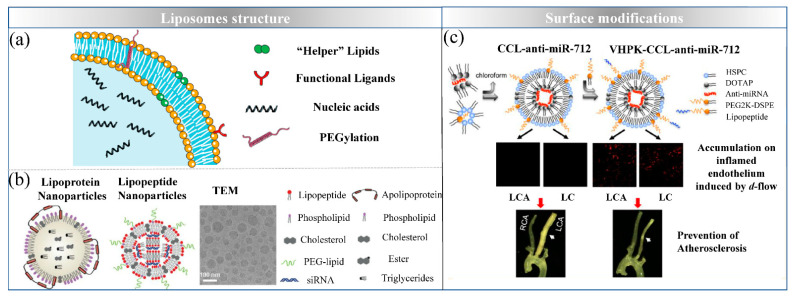
The characterization of liposomes. (**a**) The structure of liposomes; (**b**) The lipopeptide nanomaterials for siRNA delivery with the more efficient selectivity of delivery to hepatocytes (reproduced with permission from copyright 2014 National Academy of Sciences); (**c**) modification strategies for targeted delivery in atherosclerotic lesions (reproduced with permission from copyright 2015 American Chemical Society).

As shown in Figure 4, severals methods for the development of stable lipoplexes are available, which are mostly used in combination with “helper” lipids to promote the conversion of a lamellar structure to a non-lamellar phase at low pH, owing to the geometry of the helper lipid and the formation of hydrogen bonds. For example, cholesterol (CHOL) often used as a helper lipid that combines in the hydrophobic acyl chain region in the lipid bilayer to decrease lipid molecules exchange with circulating cells and increase the transfection efficiency in vivo. Additionally, other materials like DOPE and nonionic surfactants have been shown to be effective helper lipids for miRNA fusion and cytosolic release [97].

To enhance the half-life and decrease the immune response, PEGylated lipids have been introduced into liposome formulations, examples include PEG, PEG-C-DMA and mPEG-DSPE. In numerous studies, in order to sterically enhance stabilization to the liposomes, PEG (MW: 2000, 3–5 mol%) has been introduced as a polymer chain conjugated to phosphatidylethanolamine lipid [98]. However, it has been reported that repeated injection of PEGylated liposomes causes shorter half-life and hampers cellular uptake [99]. Therefore, this modification might trigger and hamper their successful application in clinical settings. Besides, solid lipid nanoparticles (SLNs) have been investigated for drug delivery owing to the non-toxic solid lipid materials, such as phosphatidylcholine, stearic acid and triacylglycerol. NA can be loaded into the core of SLNs to form electrostatic complexes by adding cationic lipids. For example, siRNA/DOTAP complexes can be encapsulated into lipid core which contain phosphatidylcholine and PEGylated lipid. This method was shown to be able to control NA release over 10 days in vivo and prolong the circulation time of NA to achieve the effect of continuous treatment.

Ligands modification onto the surface of NPs is one of the attractive approaches to improve NA targeted delivery efficacy. These ligands include peptide sequences, specific antibodies and aptamers are actively target to cell receptors or specifically surface biomarkers at disease sites. Zhi and his colleagues have designed anti-cardiac troponin I antibody modified liposomes for targeted delivery of anti-miR-1 antisense oligonucleotides (AMO-1) to relieve arrhythmogenesis [100]. This modification improved AMO-1 targeted to ischemic myocardium tissues and enhanced the curative effect. In addition, Azadeh et al. have created VHPK peptide decorated cationic lipoparticles with anti-miR-712 for atherosclerosis treatment. This modification effectively linked to vascular cell adhesion molecule 1 which on the endothelial surface in atherosclerotic lesions.

### 4.2. Preparation of Lipid-Based Delivery Systems

Given the hydrophilic nature of miRNAs, several methods have been developed for miRNA-loaded liposome preparation, each influencing liposomes properties including size, equality and EE [94]. Reverse phase evaporation is the most preferred techniques for loading miRNA. The miRNA is dissolved in aqueous solution, and lipid materials are dissolved in organic solvents, then the organic solvent is slowly removed under vacuum, and a gel phase is formed. Further evaporation of the organic solvent results in high entrapment of miRNA in the internal core of liposomes. However, this method is unsuitable for large-scale manufacture, owing to the complex process. Besides, ethanol injection is a simple method to perform, in which the injection of ethanol dissolved in an aqueous phase below a critical concentration forces the dissolved phospholipids to self-assemble in the aqueous phase and form liposomes. Microfluidics is a novel method for liposomes self-assembly supported by a microfluidic chip device with a mixture of lipids dissolved in ethanol and an aqueous solution of miRNA at a constant flow rate. Excess solvents and unbound miRNAs can be removed with dialysis.

In addition, a uniform size distribution of liposomes is an important parameter for application, which affects various properties such as EE, miRNAs release, cellular uptake, biodistribution and stability during long-term storage [95]. The methods for size reduction and ensuring uniform liposome sizes include membrane extrusion, homogenization, freeze-thaw and sonication or ultrasonic irradiation. Recently, Ong et al. compared the efficiency of different nanosizing techniques [96]. The results showed that extrusion, the most efficient technique to produce uniform liposome size, is also affected by the flow rate of the extrusion process.

### 4.3. Liposomes Delivering miRNAs for MetS Treatment

Liposomes are one of the most successful delivery systems, which can overcome certain NA limitations, such as instability in serum, the constraint of endosomal escape or cytosolic release. Additionally, liposomes-based NA delivery are available for MetS treatment. Ischemic arrhythmia caused by myocardial infarction is one of the global public health problems with high morbidity and mortality. It has been reported that local injection of anti-miR-1 antisense oligonucleotides (AMO-1) into ischemia myocardial tissue can relieve the syndrome of ischemic arrhythmia. However, it is not achievable to inject therapeutics into myocardium in the clinical. To overcome such limitations, Zhi and his colleagues designed anti-cardiac troponin I antibody modified liposomes to deliver AMO-1 to ischemic myocardium tissues. In addition, liver is the most important metabolic organ and is central to lipid transport. Therefore, lipoproteins are particularly advantageous for some liver delivery applications, owing to their high delivery efficiency and specificity. Dong et al. have developed lipopeptide nanomaterials for siRNA delivery, thus, allowing for efficient selectivity of delivery to hepatocytes without any ligand conjugation [101]. In one recent study, CHOL-PGEA conjugated liposomes was found to efficiently condense miRNA for preventing cardiac hypertrophy, because of the abundant hydrophilic hydroxyl groups in CHO-PGEA are beneficial for biocompatibility [102].

### 4.4. Challenges in Lipid-Based Delivery System

Up to now, lipid-based delivery systems have been widely studied and applied in clinical treatment due to their excellent biocompatibility and delivery efficiency. There have been 2099 clinical studies involving liposomes (searched on clinicaltrials.gov in July 2021) for various diseases treatment. Besides intravenous administration, liposomes could be administered orally which absorbed by blood across the extreme conditions of the gastrointestinal tract, including gastric acid and bile salts. Unfortunately, compared with other nanocarriers, the complicated preparation process of liposomes increases the difficulty of mass production and the heterogeneity between batches is another big challenge. Advanced techniques and manufacture processes still need to be developed continuously to support scale-independent manufacture and translate from preclinical studies to clinical use.

## 5. Polymeric Nanoparticles: Biodegradable Polymer Based Nanoformulations

With the development of nanoplatforms, the combination of polymer science and nanotechnology has brought new dimensions to the field of NA delivery. Biodegradable polymer nanomaterials are widely used in drug delivery because they efficiently deliver cargo at the target site and have favorable biocompatibility, bioavailability, safety, permeability, retention time. The size of polymeric nanoparticles for drug delivery should be considered within 50–300 nm, which are made up of the form of nanomicelles, polymersomes, nanocapsules, dendrimers and nanogels.

### 5.1. Polymeric Materials

In general, several excellent biocompatible and biodegradable polymers have been widely used as nanocarriers, which are classified into two main categories, synthetic and natural polymers. For example, poly(ethylene imine)s (PEIs) are an excellent biocompatibility synthetic polymers, which showed efficient NA encapsulation and delivery, owing to the positively charged polymer chains binding to NA, thus, forming stable complexes through electrostatic interaction [103]. In addition, PLGA is another kind of synthetic polymers which is usually combined with some cationic compounds to enhance EE. It has been reported that PLGA has favorable biocompatibility and safety, owing to degrading through hydrolysis and then enters into the Krebs cycle [104]. Natural polymers are widely used in NA delivery because the materials can be obtained from the nature, and show biodegradability, biocompatibility and low-toxicity [105]. For example, chitosan (CS) is the most widely used natural polymer because of its easy surface modification, high biocompatibility and non-cytotoxicity [106]. The positively charged amino groups of CS result in high EE, but inefficient release of the complex in cytoplasm [107]. To overcome this limitation, hydrophobic moieties or negatively charged components have been conjugated to CS to facilitate NA release [108].

### 5.2. Functionalized Strategies of Polymeric Nanoparticles

In numerous studies, polymer hybridization with liposomes displayed high stability, thus, increasing the EE and achieving more efficient delivery of NA in vivo. Zhang et al. developed nanoparticles to deliver siRNA, encapsulated within a core composed of PLGA and DOTAP [109]. The PLGA/DOTAP core ensures high EE of siRNA, with the aid of a large cavity and the ability of positively charged DOTAP o interact with the negatively charged siRNA inside the water core. DOTAP improves endo/lysosomal escape through its fusogenic properties or proton sponge effect. In addition, Yu et al. developed composite nanoparticles with PEI and anionic liposomes for siRNA and glycyrrhizic acid delivery, which not only increasing the solubility and anti-cancer effects of glycyrrhizic acid, but also decreasing the toxicity of PEI-siRNA [110]. Recently, a functionalized strategies has been developed with the hybridization chain reaction (HCR) of DNA hairpins in a polymeric nanoframework for precise siRNA delivery [111]. Based on the potential energy and a cascade HCR of DNA hairpins, the polymeric nanoframework provided stability and achieved efficient siRNA loading.

**Figure 5:**
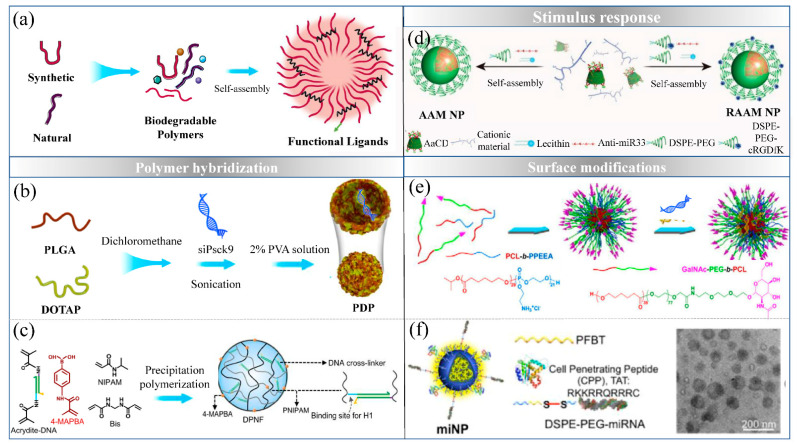
Functionalized strategies for polymeric delivery system. (**a**) The structure of polymeric delivery system; (**b**) NPs with PLGA and DOTAP hybridization (reproduced with permission from copyright 2020 American Chemical Society); (**c**) DNA nanotechnology-based strategy via the cascade hybridization chain reaction (HCR) of DNA hairpins in polymeric nanoframework (reproduced with permission from copyright 2021 Springer Nature limited); (**d**) biocompatible pH-responsive NPs synthesized by acetylation of cyclodextrins (CDs) and their polymers (reproduced with permission from copyright 2020 Wiley-VCH); (**e**) N-acetylgalactosamine functionalized mixed micellar NPs (Reproduced with permission from copyright 2012 Elsevier B.V.); (**f**) polymeric NPs as miRNA carriers together with a shear-thinning hydrogel to encapsulate the miNPs (reproduced with permission from copyright 2019 American Chemical Society).

To achieve targeted delivery, methods for some ligands conjugated onto the surfaces of NPs have been introduced above (Figure 5). For example, the N-acetylgalactosamine (GalNAc) group is effective in delivery into hepatocytes by binding with asialoglycoprotein receptor. Wang et al. described GalNAc functionalized mixed micellar NPs, which can efficiently achieve liver targeting siRNA delivery [112]. With the development of nanotechnology, precise targeted delivery based on the stimulus–response related to disease microenvironment enable lower cytotoxicity and better curative effect of NPs in vivo. Strategies for pH-responsive delivery systems based on the acidic microenvironment of atherosclerosis with inflammation serves as effective platforms for NA release. Li et al. developed a pH-responsive delivery system synthesized by acetylation of cyclodextrins (CDs) and their polymers, which targeted deliver anti-miR-33 in the inflamed atherosclerotic lesions [113].

### 5.3. Polymeric Delivery of miRNAs for MetS Therapy

Many natural and synthetic polymers have been widely used as nanocarriers, owing to the potential advantages of NA protection, biodegradability or controlled release. As shown in Table 2, one group has recently developed polymeric NPs made with a cross-linked chitosan polysaccharide polymer, which has been found to protect and delivery miR-33 to macrophages, where they regulate cholesterol efflux and prevent of lipid accumulation and atherosclerosis [114]. Bejerano et al. developed hyaluronan-sulfate NPs mediated by calcium ion bridges to deliver miRNA-21 mimics to cardiac macrophages, thereby reducing inflammation and improving cardiac healing [115]. Yang et al. developed a delivery system using cross-linked elastin-like protein−HA formed a shear-thinning injectable hydrogel to carry miR-199a-3p for localized delivery, thus, significantly improving cardiac function, decreasing scar size and enhancing capillary density [116].

To achieve high efficiency and accuracy targeting, some functionalized ligands have been introduced to greatly enhance NPs uptake and targeted delivery. Wang et al. developed a promising liver targeting siRNA delivery system based on N-acetylgalactosamine functionalized mixed micellar nanoparticles (Gal-MNP), which can efficiently deliver siRNA to hepatocytes and silence the target gene expression after systemic administration. The Gal-MNP were assembled in aqueous solution from mixed N-acetylgalactosamine functionalized poly(ethylene glycol)-b-poly(ε-caprolactone) and cationic poly(ε-caprolactone)-b-poly(2-aminoethyl ethylene phosphate) (PCL-b-PPEEA). Systemic delivery of Gal-MNP/siRNA did not induce the innate immune response or positive hepatotoxicity.

### 5.4. Challenges in Polymeric Delivery System

In recent decades, high EE, non-toxicity and control release effects of biopolymers make them an appropriate choice for drug delivery. Additionally, the delivery system, using natural and synthetic biopolymers, has expanded the possibility of many routes for novel delivery. To date, researches of oral, nasal and transdermal means of drugs delivery have been explored to increase patient compliance. Still, there is room to explore more biodegradable polymers for drugs delivery. It should be noted that the invention of novel biomaterials and functionalized design are complex tasks that requires detailed understanding of the interactions between polymers and the biological environments as well as the effectiveness and safety for clinical applications.

## 6. Emerging Novel Delivery Systems

### 6.1. Inorganic NPs

**Figure 6:**
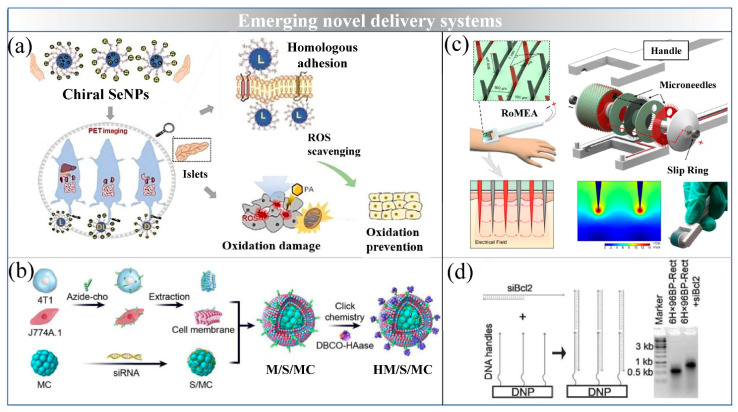
Emerging novel delivery systems for NA delivery. (**a**) L-Se-NPs increased affinity for the cell membrane as well as protected pancreas from oxidative damage (reproduced with permission from copyright 2019 Wiley-VCH); (**b**) magnetosomes which decorated with camouflaged chimeric membrane (reproduced with permission from copyright 2020 Elsevier B.V); (**c**) schematic illustration of rolling microneedle electrode array for siRNA delivery (reproduced with permission from copyright 2020 Elsevier Ltd.); (**d**) loading mechanism of siRNA onto DNA origami (reproduced with permission from copyright 2017 Wiley-VCH).

Calcium phosphate (CaP) is the main inorganic component of hard tissues, such as bones and teeth, in vertebrates, and the synthetic calcium phosphate nanomaterials have high biocompatibility and biodegradability for NA delivery. As shown in Figure 6, Mauro et al. developed bioinspired and negatively surface charged CaP-NPs, which are able to encapsulate and carry miRNAs into cardiac cells for the treatment of cardiovascular disease both in vitro and in vivo [124].

Selenium (Se) is an essential trace element for the human body, which synthesize selenoprotein and participate in physiological reactions [125]. Se-NPs have been proposed as more efficient inorganic delivery system with drug-carrying synergistic functions than other inorganic nanomaterials, owing to the anti-oxidation functions of Se. Zheng et al. developed an inorganic delivery system based on co-delivery of Se and siRNAs to drug-resistant tumor cells in vitro [126]. Layered double hydroxide nanoparticles-supported Se-NPs have been compacted with siRNAs via electrostatic interactions, thus, protecting the siRNA from degradation, these NPs have shown excellent ability to deliver siRNA into cells, including enhancing siRNA internalization and promoting siRNA escape from endosomes. In addition, Huang et al. investigated the fusion of chiral Se-NPs to the cell membrane and their protection activity against palmitic acid induced oxidative damage of islet β cells. L-Se-NPs increase the affinity for the cell membrane and exhibit preferential accumulation in the liver, spleen and pancreas, thus, enabling NA delivery in the treatment of MetS [127].

### 6.2. Magnetosomes

Magnetosomes are an emerging magnetic nanocrystals surrounded by a phospholipid bilayer, which secreted by magnetosome-producing microorganisms [128]. Because of their special properties such as a single magnetic domain, the size of the nanostructure, excellent biocompatibility, low toxicity, surface modification and the ability for control by an external magnetic field, magnetosomes are an ideal target for drug carriers [129]. Hypoxia-inducible factor-1 (HIF-1) siRNA has been successfully loaded onto purified bacterial magnetosomes and shown showed excellent antitumor activity [130]. Magnetism-mediated targeting with MRI guidance enhances siRNA accumulation at tumor sites; in addition, cytoplasmic trafficking of siRNA via membrane fusion enables efficient silencing of HIF-1. Therefore, miRNA adsorption by magnetosomes, with satisfactory superparamagnetism, high magnetization and a positive charge, is feasible for the treatment of MetS. However, the cumbersome cultivation process and low yield have hindered the application of magnetosomes. Improved methods for producing strains from existing high-yield magnetosomes or isolating many new magnetosome-producing microorganisms will be a primary future objective.

### 6.3. DNA Origami

Based on structural DNA nanotechnology, DNA origami is one of the emerging novel delivery systems with precisely nanoscale shapes. DNA origami is self-assembled to form defined arbitrary shapes by a long single strand of DNA (scaffolding strands) and hundreds of short DNA strands (helper strands) [131]. Recent studies have investigated different structures of DNA (triangle, tube and square) as effective delivery carriers for siRNA, either in vitro or in vivo [132]. The biodistribution results have demonstrated that DNA origami can target the liver, owing to the reticuloendothelial system, thus, indicating potential for the treatment of NAFLD and NASH.

### 6.4. Microneedles

Biomacromolecular drugs, including proteins, polysaccharides and NA, are widely used in MetS treatment. However, the delivery of biomacromolecular drugs in vivo still faces many obstacles. For example, the most commonly used administration method is intravenous or intramuscular injection. Frequent injection causes discomfort, pain and fear in patients, thus resulting in poor compliance. Microneedles is one of the emerging novel delivery systems through administration of surface skin. Microneedle patches typically consist of many microneedle arrays with 500–800 mm in height and made of water-soluble or biodegradable polymers, which overcome epidermis barriers and allow direct placement of therapeutic agents with minimal invasiveness [133]. Some studies have developed microneedle patches to deliver an anti-obesity drugs to the subcutaneous white AT [133,134]. To the best of our knowledge, no studies have used NA delivery via microneedle patches for MetS treatment to date. To implement an efficient and extensive siRNA therapy in vivo, one recent study has reported a rolling microneedle electrode array (RoMEA), which uses parallel circular blades with microneedle arrays on edge as electrodes [135]. RoMEA integrates closely-spaced microneedle electrodes and a rolling structure to allow for low-damage and large-area siRNA transfection. After application of RoMEA, regular micropores are established, thus, enabling efficient siRNA delivery for the treatment of cancer. However, the immunogenic profiles and pharmacological mechanisms of microneedles must be carefully evaluated for clinical settings.

## 7. Nanosystem-Based Delivery of miRNAs for MetS Treatment in Clinical Transformation

MiRNA-based therapeutics have exerted great effects for various diseases, including cancer, neurodegenerative diseases and enteritis. However, many studies for MetS treatment were forced to suspend due to the serious adverse effects or failure of therapy under the complicated microenvironments. MetS requires a long-term effort for the successful treatment because a number of complications would appear in the development stage. To date, the only miRNA-based agent for MetS treatment, known as RG-125/AZD4076, have reached phase I clinical trials. This agent could increase insulin signaling and regulate adipocyte size by silencing of miR-103 and miR107, and then decrease the levels of NAFLD and NASH [86,87]. With the development of nanotechnologies, nanomaterials have shown great potential in drug delivery and gene therapy from organic nanomaterials to inorganic nanoparticles. In order to accelerate clinical transformation, further optimization of nanosystem-based delivery is required tuned to their clinical applications. Areas for further improvement are (1) increasing the circulation time of nanocarriers in body to achieve sustainable treatment after drugs withdrawal, and (2) expanding the targets of nanomedicine to repair the complications of Mets, and (3) understanding of the interactions between nanocarriers and the biological environments to decrease toxicity in vivo, and (4) optimizing production process to support scale-up in industry. At last, it may be expected that nanosystem-based delivery of miRNAs is the perfect choice as next-generation therapeutics.

## 8. Concluding Remarks and Future Perspectives

To date, the development of nanotechnology has shown promising efficacy in animal models to achieve more effective delivery of NA addressing MetS. This review provides insights into current treatments of miRNA for MetS, with a focus on the development of functionalized nanocarriers for enhanced NA delivery, and suggest several future directions including NA modification, combinatory therapy in the same nanotech delivery vehicle, delivery route choice and biomimetic strategies.

The major obstacles for NA application is the instability of short-stranded nucleic acid, owing to the abundance of nucleases. In efforts to address the shortcoming, NA modifications have been proposed on the internucleotidic-linkage and sugar moiety, thus, leading to superior stability, safety and efficacy of miRNAs. 2′-O-modification, a natural modification of small RNA after transcription, increases the Tm of the RNA and improve the binding affinity to the target genes [136]. In addition, a new type of nucleotide analogue known as locked nucleic acids (LNA) has been widely used to improve binding affinity and achieve high bio/thermostability, as compared with conventional DNA or RNA [137]. Another modification of the bridge structure within the nucleotide junction, producing peptide nucleic acids (PNA), has involved replacement of amide 2-aminoethylglycine bond of the neutral peptide chain in the phosphate backbone. Because PNA has no negative charge, the stability and specificity of binding to NAs are greatly improved [138].

In terms of cancer treatment, doxorubicin (DOX) is a widely used and effective nano-medicine. However, frequent injection and high doses of DOX lead to tumor tolerance to chemotherapy and cytotoxicity [139]. Combined NA and DOX co-delivery not only decreases the risk of chemotherapy resistance but also achieves excellent superadditive effects. For example, Wang et al. have developed an assembled delivery platform for co-delivery of miRNA and chemotherapy to achieve the synergistic effects. This system was fabricated by conjugation of microRNA-31 onto doxorubicin-loaded mesoporous silica nanoparticles with a poly(ethyleneimine)/HA coating, and drug release was triggered by the acidic environment of tumors [140]. For the treatment of MetS, some classes of antidiabetic medication show adverse effects in the clinic (Figure 1). Therefore, a co-delivery system combining NA with drugs has enabled cooperative inhibition of MetS development and provided potential synergistic or combined effects with different mechanisms, in addition to yielding benefits including diminished doses and toxicity.

Most studies have focused on the design and development of effective NA delivery systems for the treatment of MetS. Some studies have greatly contributed to clinical practice and application [141,142]. Oral administration, which is convenient, cost-effective and supports patient compliance, is considered the best route for gene delivery and attracted extensive attention [143]. Several oral delivery systems for genes based on NPs have demonstrated anti-inflammation efficacy and applications in tumor therapeutics [143,144]. However, to our knowledge, no studies involving oral miRNA delivery systems for MetS treatment have been reported to date. The rapid degradation by nucleases and harsh GI environment severely limits their in vivo applications, particularly for oral delivery. Recent studies have shown that the mechanism of dietary miRNAs absorbed through the stomach, which highlights the development of small RNA therapeutics through oral delivery [145]. This finding indicates a major mechanism underlying the absorption of dietary miRNAs, thus, uncovering an unexpected role of the stomach and shedding light on the development of small RNA therapeutics through oral delivery.

More recently, cell membrane-coated nanoparticles with biomimetic surface composition, shape and movement with respect to normal cellular physiology have been developed. Various types of membranes have been used to fabricate biomimetic nanoparticles, such as bacteria, cancer cells, platelets, red blood cells, stem cells and leukocytes. The cell membrane-coated nanoparticles have high biocompatibility and prolonged half-life in the circulation, and exhibit specific targeting. Recently, Zhang et al. have developed a novel type of artificial platelets with biomimetic properties, long circulation and liver targeting, which enable efficient delivery of siRNA for the treatment of hypercholesterolemia. Although the technology of cell membranes coating has not yet achieved full clinical implementation, its clear advantages and the abundant sources of cell membrane offer a solid foundation for its industrial production and implementation in individual precision medicine approaches.

These nanoscale delivery systems provide excellent targeting and specificity through rational design and surface modification. However, it is urgent need to fully understand the interactions between nanomaterials and biological systems and to overcome challenges for clinical translation. Discovering the fate of NPs after administration and to achieve scale-up in industry will be crucial.

## Figures and Tables

**Table 2:** Developments in delivery nanosystems of miRNAs for therapy MetS.

NA	Nanocarrier Classification	Composition and Feature	Disease	Target Strategies	References
siPPARA	Exosomes	Exosomes isolated from plasma	Obesity	-	[93]
miR-21 mimic and inhibitor	Exosomes	Peripheral blood-derived exosomes	MI	-	[91]
cholesterol-modified miR-210	Exosomes	Conjugated c(RGDyK) peptide on mesenchymal stromal cell (MSC)-derived exosomes	Cerebral ischemia	Peptide target Ischemia part	[90]
miR-155 mimic	Exosomes	ATM-derived exosomes	IR	-	[92]
miR-192-5p inhibitor	Exosomes	Exosomes isolated from serum	NAFLD	-	[117]
miR-103/107 antagomirs	Liposomes	Liposomes were composed of DLin-KC2-DMA, distearoyl phosphatidylcholine, cholesterol and mPEG2000-DMG, used at the molar ratio 50:10:38.5:1.5	T2DM	-	[118]
miR-106b, miR-148b, and miR-204 mimics	Liposomes	Cationic lipid: DOTAP	MI	-	[119]
anti-miR-712	Liposomes	DOTAP: DSPE-PEG2k:HSPC:chol (9.3:3.1:52.6:35, molar ratio)	AS	-	[120]
anti-miR-1 antisense oligonucleotides	Liposomes	anti-cardiac troponin I (cTnI) antibody modified liposomes (EPC, CHO and DSPE-PEG2000, with molar ratio of 49/50/1)	Ischemic myocardium	-	[100]
miR-182 inhibitor	Liposomes	CHO/PGEA	Cardiac hypertrophy	-	[102]
siFVII	Liposomes	DSPC, PEG-lipid, CHO	Liver disease	-	[101]
siFVII	Liposomes	N-acetylgalactosamine (GalNAc)–PEG–lipid	Liver disease	Receptor target liver	[121]
miR-33 mimic	Polymers	Chitosan via the ionic gelation method using TPP as a cross-linker	AS	Inflammation	[114]
anti-miR33	Polymers	pH-responsive polymers synthesized by acetylation of cyclo-dextrins (CDs) and DSPE-PEG	AS	pH-response	[122]
miR-199a-3p mimic	Polymers	a core−shell structure: PFBT core, PEG shell, and TAT conjugate on the surface of NPs	MI	-	[116]
miR-21 mimic	Polymers	spontaneously assembled due to the complexation of hyaluronan-sulfate with the NA mediated by calcium ion bridges	MI	-	[115]
sipcsk9	Polymers	PLGA: DOTAP (ratio of 20:3), platelets membrane coating	Hypercholesterolemia	-	[109]
siapoB	Polymers	The micellar NPs assembled in aqueous solution from mixed N-acetylgalactosamine (GalNAc) functionalized PCL-b-PPEEA	Liver disease	Receptor target liver	[123]
miR-133a	Inorganic nanomaterials	negatively charged calcium phosphate nanoparticles	CVD	-	[124]

## Data Availability

Not applicable.

## Abbreviations

MetS: metabolic syndrome; MiRNAs: microRNAs; NA: nucleic acids; CVD: cardiovascular disease; T2DM: type 2 diabetes mellitus; RES: reticuloendothelial system; cmiRNAs: circulating miRNAs; AT: adipose tissue; IR: insulin resistance; PDK1: 3-phosphoinositide dependent kinase-1; NAFLD: nonalcoholic fatty liver disease; NASH: nonalcoholic steatohepatitis; MSCs: mesenchymal stem cells; TEM: transmission electron microscopy; DLS: dynamic light scattering; ATM-Exos: adipose tissue macrophages exosomes; VAT-Exos: visceral adipose tissue derived exosomes; BBB: blood−brain barrier; EE: encapsulation efficiency; CHOL: cholesterol; AMO: antisense oligonucleotides; VCAM1: vascular cell adhesion molecule 1; CAD: coronary heart disease; PCSK9: proprotein convertase subtilisin/kexin type 9; LDLR: low density lipoprotein receptor; CS: chitosan; HA: hyaluronic acid; HCR: hybridization chain reaction; GalNAc: N-acetylgalactosamine; LDL-c: low-density lipoprotein cholesterol; ASGR: asialoglycoprotein receptor; CDs: cyclodextrins; CaP: calcium phosphate; Se: selenium; HIF-1: hypoxia-inducible factor-1; RoMEA: rolling microneedle electrode array; LNA: locked nucleic acids; PNA: peptide nucleic acids; DOX: doxorubicin.
